# Is NMDA-Receptor-Mediated Oxidative Stress in Mitochondria of Peripheral Tissues the Essential Factor in the Pathogenesis of Hepatic Encephalopathy?

**DOI:** 10.3390/jcm11030827

**Published:** 2022-02-04

**Authors:** Elena Kosenko, Lyudmila Tikhonova, Gubidat Alilova, Carmina Montoliu

**Affiliations:** 1 Institute of Theoretical and Experimental Biophysics of Russian Academy of Sciences, 142290 Pushchino, Russia; ljudasik09@rambler.ru (L.T.); hells2012@yandex.ru (G.A.); 2 Hospital Clinico Research Foundation, INCLIVA Health Research Institute, 46010 Valencia, Spain; 3 Pathology Department, Faculty of Medicine, University of Valencia, 46010 Valencia, Spain

**Keywords:** hepatic encephalopathy, hyperammonemia, NMDA receptors, superoxide radical, hydrogen peroxide, antioxidant enzymes, oxidative stress, mitochondria, liver, heart, pancreas, erythrocytes

## Abstract

Background: Hepatic encephalopathy (HE) is a neuropsychiatric syndrome of increased ammonia-mediated brain dysfunction caused by impaired hepatic detoxification or when the blood bypasses the liver. Ammonia-activated signal transduction pathways of hyperactivated NMDA receptors (NMDAR) are shown to trigger a cascade of pathological reactions in the brain, leading to oxidative stress. NMDARs outside the brain are widely distributed in peripheral tissues, including the liver, heart, pancreas, and erythrocytes. To determine the contribution of these receptors to ammonia-induced oxidative stress in peripheral tissues, it is relevant to investigate if there are any ammonia-related changes in antioxidant enzymes and free radical formation and whether blockade of NMDARs prevents these changes. Methods: Hyperammonemia was induced in rats by ammonium acetate injection. Oxidative stress was measured as changes in antioxidant enzyme activities and O_2_^•−^ and H_2_O_2_ production by mitochondria isolated from the tissues and cells mentioned above. The effects of the NMDAR antagonist MK-801 on oxidative stress markers and on tissue ammonia levels were evaluated. Results: Increased ammonia levels in erythrocytes and mitochondria isolated from the liver, pancreas, and heart of hyperammonemic rats are shown to cause tissue-specific oxidative stress, which is prevented completely (or partially in erythrocyte) by MK-801. Conclusions: These results support the view that the pathogenesis of HE is multifactorial and that ammonia-induced multiorgan oxidative stress-mediated by activation of NMDAR is an integral part of the disease and, therefore, the toxic effects of ammonia in HE may be more global than initially expected.

## 1. Introduction

Hepatic encephalopathy (HE) is a neuropsychiatric disorder that develops in patients with severe liver dysfunction. Clinical symptoms of the pathology range from minimal changes in intellectual function to dementia and coma, with often fatal outcomes [1].

Although “hepatic encephalopathy”, as a medical term of the disease, has been in use for over a century [2] and conceptually reflects the relationship between liver damage and brain pathology, the pathogenic mechanisms of dysfunction of the brain in HE remain unclear.

However, elevated blood ammonia (hyperammonemia (HA)) caused by a combination of impaired detoxification by the liver due to hepatocytes damage and portosystemic shunting, which allows ammonia to enter the brain unhindered, is considered the main contributing factor to ammonia accumulation in the brain, and its toxic effect is responsible for both cerebral dysfunction and clinical symptoms of HE [3,4,5].

The precise pathophysiologic mechanisms of encephalopathy in hyperammonemic conditions are not completely understood, but ammonia is believed to affect brain cells via several mechanisms [6,7].

Ammonia has been found to disrupt brain energy metabolism [8], aerobic glucose oxidation [9,10], mitochondrial respiratory chain, and oxidative phosphorylation, thereby decreasing ATP synthesis [11]. Additionally, ammonia remarkably enhances the generation of reactive oxygen and nitrogen species [12,13], inhibits antioxidant enzymes and glutamine synthetase resulting in oxidative stress [14,15,16], and blocks ammonia transformation into the relatively non-toxic glutamine [13,17,18].

Furthermore, our previous studies have shown that both global disruption of vital biochemical processes in the brain and the death of animals with acute hyperammonemia were nearly completely prevented by different antagonists of glutamate NMDA receptors (NMDARs), particularly by administration of MK-801, a potent non-competitive receptor antagonist [6]. These findings confirm a key role of the glutamate as the main neurotransmitter, which induces a cascade of pathological reactions in hyperammonemic conditions, primarily due to hyperactivation of NMDAR, ultimately leading to cerebral dysfunction [13,18,19,20,21,22,23] and neurological manifestations.

However, the recent discovery of functional NMDARs outside the CNS in various peripheral tissues [24,25,26,27] suggested that multiorgan failure may be an integral part of the disease [28], and the toxic effects of ammonia, mediated by NMDAR, in HE may be broader and more harmful than was previously believed [25].

It is well known that there is a direct relationship between a degree of hepatic damage and pancreatic injury in patients with chronic liver disease [29,30]. Chronic hepatic disease also affects cardiovascular functions [31]. Cirrhotic cardiomyopathy, in turn, is associated with a decreased arterial oxygen saturation [32] and oxygen levels in the tissues, resulting in impaired cardiac aerobic energy metabolism [33]. Most patients develop anemia by various mechanisms [34,35], and this pathology is accompanied by a broad range of abnormalities in hematological parameters [36,37], including reduction in red blood cell count [38], hemoglobin and hematocrit values, and appearance of erythrocytes with atypical morphology [39] and other abnormalities [40]. This ultimately leads to a decrease in the oxygen-carrying capacity of blood [41], which, in turn, can result in multiple organ hypoxia [42].

Therefore, HE is supposedly multifactorial in its etiology, including the factor of pre-existing raised levels of blood and brain ammonia, because of the impaired liver’s detoxification pathways and toxicity of systemic ammonia that has a deleterious effect on brain cells and most likely on other peripheral organs [43]. However, as yet there is insufficient information to draw conclusions about non-neuronal organs as sensitive targets of ammonia toxicity in experimental animals, and even in HE patients. To our knowledge, the problem has not been fully explored, although the concept of the leading role of ammonia in the development of multiorgan failure is far from new [44].

Furthermore, there is little evidence of both accumulation and possible mechanisms of toxic effects of ammonia in non-neuronal tissues [44]. Given this, our research efforts narrow the focus to study ammonia distribution in peripheral tissues containing NMDARs and whether the toxic effects of ammonia are mediated by these receptors.

Oxidative stress has been shown to be one of the deleterious processes in the pathological cascade triggered by hyperactivation of NMDAR in the brain of hyperammonemic animals [15,22,45,46] and patients with HE [47,48]. Nevertheless, the exact relationship between NMDA receptors present in peripheral non-neuronal tissues and oxidative stress involved in the pathogenesis of multiorgan pathology in HE [28,49] is yet to be fully explained.

The aim of this study was to evaluate the effects of NMDAR blockade with MK-801 on the imbalance between oxidant and antioxidant systems leading to oxidative stress, focusing on superoxide radical and hydrogen peroxide production by mitochondria isolated from NMDAR-equipped non-neuronal tissues including the pancreas, heart, and liver of hyperammonemic rats. Antioxidant enzyme activities of glutathione peroxidase, glutathione reductase, catalase, and superoxide dismutase in mitochondria from tissue and in erythrocytes were also measured.

To reveal the role of NMDARs, the potent non-competitive NMDAR antagonist MK-801 ((5S,10R)-(+)-5-Methyl-10,11-dihydro-5H-dibenzo[a,d]cyclohepten-5,10-imine hydrogen maleate), an extensively investigated drug with pleiotropic physiological effects many of which involve the CNS [50] was used at a dose able to protect brain cells from glutamate excitotoxicity in a condition known as ammonia-activated oxidative stress [51].

In an animal model, ammonia concentrations in the blood increased to levels found in some human pathologies accompanied by rapid neurologic complications [52,53]. This highlights the need for further investigation of the mechanisms underlying the initial phases of ammonia-induced damage.

## 2. Materials and Methods

### 2.1. Materials

MK-801, EDTA, EGTA, DL-dithiothreitol (DTT), nitrotetrazolium blue (NTB), scopoletin, horseradish peroxidase (HRP), phenylmethylsulfonyl fluoride (PMSF), leupeptin, pepstatin, aprotinin, Percoll, bovine serum albumin, flavin adenine dinucleotide, flavin mononucleotide, NADPH, glutathione (GSH), xanthine, xanthine oxidase, sucrose, Tris, catalase, superoxide dismutase (SOD), and glutathione reductase (GR) were obtained from Sigma Chemical Co. (St. Louis, MO, USA). Other reagents were of the highest grade available.

### 2.2. Experimental Section

#### 2.2.1. Experimental Design

Our studies were carried out in accordance with ethical principles set out in the Helsinki Declaration for the care and use of laboratory animals and in compliance with EU legislation Directive 2010/63/EU) and with the Order of the Ministry of Health of the Russian Federation of 19.06.2003 № 267 “Regulations in Laboratory Practices”.

#### 2.2.2. Animals

For each experiment, eight male Wistar rats weighing 210–230 g were randomly divided into groups. The animals were housed in a vivarium, at a stock density of four per split-level cage (40 × 30 × 20 cm) at room temperature under a natural light regime and were fed a standard laboratory chow diet and water ad libitum.

The rats in the first group (ammonia) were injected i.p. with a sublethal dose (7 mmol/kg) of ammonium acetate. As animals exhibited hyperventilation, clonic convulsion, fell into a coma, and died 20 ± 2 min after having the injection, rats were sacrificed by decapitation 15 min after injection [22], usually after experiencing two convulsive episodes [22]. This time interval was chosen based on our previous results showing that the effects of acute ammonium intoxication on brain energy metabolism can be clearly seen 15 min after injection [17].

Control rats were given saline and killed 15 min later.

The rats in the second group (MK-801 + ammonium acetate) initially received 2 mg/kg of MK-801 and then 15 min later 7 mmol/kg of ammonium acetate, and 15 min after the second injection, they were decapitated.

MK-801 alone in the above dose was administered into the rats (the third group), and animals were then killed 30 min later.

### 2.3. Preparative and Analytical Methods

#### 2.3.1. Determination of Ammonia in Plasma

Blood was drawn from the retro-orbital plexus into citrate-treated tubes. Plasma obtained by the standard method was deproteinized with a cold mixture (−20 °C) of 6% HClO_4_ and 40% ethanol (final concentration of HClO_4_ 3.5% *w*/*v*) and neutralized with 30% KOH (−20 °C) to pH 6. KClO_4_ crystals were precipitated by centrifugation, and the resulting supernatant was immediately used to determine plasma ammonium by the microfluorimetric method described by Kosenko et al. (2008) [54].

#### 2.3.2. Isolation of Mitochondria Using a Self-Generated Percoll Gradient

Pancreas, heart, and liver mitochondria were isolated by a combination of differential and self-generated Percoll-gradient centrifugation, essentially according to a protocol developed by Graham [55] except that 5 μM aprotinin, an inhibitor from a range of serine proteases, was added to isolation medium. This protocol enables a highly purified and intact preparation, as assessed by relatively low contamination of the mitochondria with other subcellular compartments (low marker enzyme activities), sufficiently high respiratory control index, and close to theoretical ADP/O ratios (phosphorylation capacity) upon oxidation of lipid and non-lipid substrates [56]. Mitochondrial protein concentration was determined by the Lowry method using bovine serum albumin as standard [57].

#### 2.3.3. Disruption of Mitochondria to Determine Enzyme Activity

For determination of glutathione reductase (GR, EC 1.6.4.2), glutathione peroxidase (GP, EC 1.11.1.9), and superoxide dismutase (SOD, EC 1.15.1.1) activities, mitochondria were disrupted by osmotic shock in two volumes of a lysis mixture (containing 10 mM potassium phosphate buffer, pH 7.4, 0.5 mM PMSF, 0.25 mM EGTA, 1 mM DTT, 10 μM leupeptin, 1.5 μM pepstatin, and 0.15 μM aprotinin) at 4 °C and submitted to three cycles of freezing and thawing. The samples were then centrifuged at 14,000× *g* for 10 min. Enzyme activity was measured in the supernatant immediately after separation.

Catalase (EC 1.11.1.6) activity was determined in undisturbed mitochondria in the presence of 1% Triton X-100.

#### 2.3.4. Determination of Enzyme Activities in Mitochondria

The enzyme activity of GR and GP was determined by the methods described earlier [51]. Activities of both enzymes were expressed as nmol/min × mg protein.

Total SOD activity was determined by Beauchamp and Fridovich’s method [58] by inhibition of NTB reduction in the presence of the xanthine–xanthine oxidase system. The mn^2+^-SOD activity was calculated as the difference between total activity (Cu^2+^,Zn^2+^-SOD plus Mn^2+^-SOD) and activity measured in the presence of the Cu^2+^,Zn^2+^-SOD inhibitor cyanide (1 mM). The enzyme activity was expressed as U/min × mg. protein. One unit of SOD activity was defined as the amount of SOD required to inhibit the NTB reduction rate by 50%.

Catalase activity was assessed in the presence of hydrogen peroxide by measuring the decrease in absorption at 240 nm, as described by Aebi [59]. The enzyme-specific activity was expressed in terms of the first-order reaction rate constant K s^−1^ per mg of protein [60].

#### 2.3.5. Determination of Enzyme Activities in Erythrocytes

Erythrocytes were obtained from animal blood taken during decapitation with subsequent purification from leukocytes and platelets by filtration through microcrystalline cellulose and α-cellulose [61]. After washing, the packed cells were lysed at 4 °C by a hypoosmotic stabilizing solution containing 10 mM triethanolamine (pH 7.5), 35 µM K^+^-EGTA, 0.7 mM mercaptoethanol, and 0.02% saponin (final concentration).

The enzyme activity of GR, GP, SOD, and catalase was measured by the methods used for enzyme determination in mitochondria (Section 2.3.4).

GR and GP activities were expressed as µmol/min × g Hb. Catalase activity was expressed in terms of the first-order reaction rate constant, s^−l^ per g of Hb and SOD as U/min × g Hb, where one unit of SOD activity was defined as the amount of SOD required to inhibit the rate of NTB reduction by 50%.

#### 2.3.6. Preparation of Acid Extracts of Erythrocytes for Determination of Ammonia Concentration

Washed erythrocytes were mixed (1:5) with the cold (−20 °C) 6% HClO_4_/40% C_2_H_5_OH solution and centrifuged at 10,000× *g* for 10 min at −10 °C. The supernatant was neutralized (+4 °C) with cold 30% KOH. Following the second centrifugation for removing KClO_4_ crystals, a clear supernatant was immediately used to determine ammonium by the microfluorimetric method described by Kosenko et al. (2008) [54].

#### 2.3.7. Preparation of Submitochondrial Particles for Determination of Superoxide Radical Production

Submitochondrial particles (SMPs) from mitochondria of different tissues were prepared, essentially as described earlier [51], and with the aim of avoiding superoxide radical (O_2_^•−^) generation in mitochondria during their destruction by ultrasonication, in our study, mitochondria were disrupted by osmotic shock and three freeze–thaw cycles, as stated above (Section 2.3.3).

SMPs without both SOD isoforms were used to measure O_2_^•−^-dependent reduction of dichlorophenolindophenol by the Forman and Kennedy method [62], as described earlier [12].

#### 2.3.8. Measurement of Hydrogen Peroxide Production in Mitochondria

H_2_O_2_ production by isolated mitochondria was monitored fluorometrically by scopoletin fluorescence in the presence of HRP, as described previously [51].

#### 2.3.9. Preparation of Protein-Free Extracts of Mitochondria for Determination of Ammonia Concentration

The analytical procedure for the preparation of protein-free extracts of mitochondria was similar to that used for the extraction of blood plasma (Section 2.3.1), except for the use of an extractable mixture consisting of 8% HClO_4_ and 40% ethanol.

The concentration of ammonia in mitochondrial extracts was assayed using the microfluorometric method described by Kosenko et al. (2008) [54].

#### 2.3.10. Statistical Analysis

The results are expressed as mean ± SEM (standard error of the mean). Statistical processing of the results was performed using the program Prizm 5.0 for Windows (GraphPad Software, San Diego, CA, USA). The normality of the distribution of variables was confirmed by the Kolmogorov–Smirnov test. Pairwise comparisons were carried out using Student’s *t*-test, and multiple comparisons were performed using one-way ANOVA, together with Bonferroni’s multiple comparison test. *p* < 0.05 was considered significant.

## 3. Results

### 3.1. MK-801 Partially Reduces Ammonia Accumulation in Plasma and Erythrocytes of Hyperammonemic Animals

**Figure 1:**
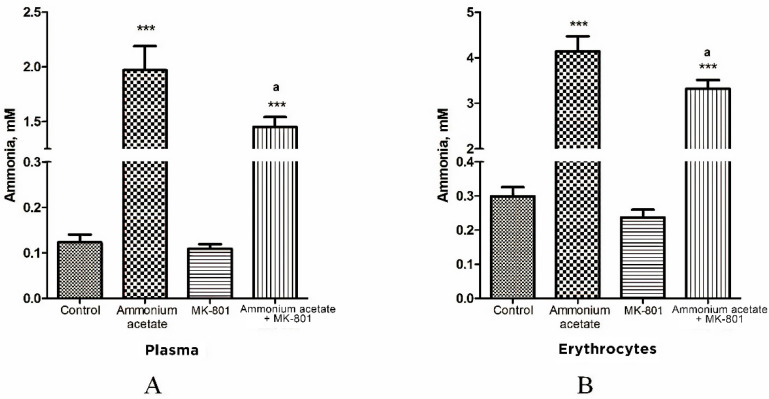
Ammonia concentration of plasma (**A**) and erythrocytes (**B**) in animals from different experimental groups. Rats (*n* = 8 per group) were injected intraperitoneally with ammonium acetate (7 mmol/kg), MK-801 alone (2 mg/kg i.p.), and MK-801 15 min before ammonium acetate injection. Rats from the control group received a saline injection. Plasma and erythrocyte ammonia concentration was determined as indicated in Materials and Methods. The results are presented as mean ± SEM. Values significantly different from the control group are designated with three (***) asterisks: *** *p* < 0.001 (Student’s *t*-test). Values significantly different from the ammonia group are designated with ^a^. ^a^ *p* < 0.05 (with the Bonferroni correction for multiple comparisons).

We showed earlier that MK-801, a potent non-competitive NMDAR antagonist, reduces excessive amounts of brain ammonia in hyperammonemic rats [19]. We then examined whether MK-801 could affect ammonia levels in NMDAR-equipped circulating erythrocytes [63,64] in rats with hyperammonemia. Plasma ammonia levels in all of the studied groups of animals were simultaneously determined (Figure 1).

In rats from control group, plasma ammonia concentration was 0.123 ± 0.017 mM, and it was significantly lower or less than that in rats injected with ammonium acetate (1.97 ± 0.22 mM (*p* < 0.001)) or MK-801 + ammonium acetate (1.45 ± 0.09 mM (*p* < 0.001)), respectively. No significant difference in plasma ammonia concentration was observed between the MK-801-treated and control groups (Figure 1A).

As shown in Figure 1B, the ammonia concentration of erythrocytes is higher than that of plasma in all examined animal groups and corresponds to the normal distribution of ammonia in the blood [65].

The ammonia content of erythrocytes in control rats was 0.299 ± 0.027 mM. After ammonium injection, the content of ammonia in the cells was significantly increased and reached 4.14 ± 0.33 mM (*p* < 0.001) and remained higher than that in control animals treated with MK-801 and ammonium acetate (3.32 ± 0.19 mM, *p* < 0.001) but decreased significantly by 19.8% (*p* < 0.05) when compared with levels in animals from ammonia group.

### 3.2. The Effect of MK-801 on Ammonia Levels in the Liver, Heart, and Pancreas Mitochondria of Hyperammonemic Animals

As increased ammonia is the main mediator in brain mitochondria dysfunction triggered by activation of NMDA receptor signaling pathways [18], we tested the ammonia content in mitochondria isolated from the liver, heart, and pancreas of hyperammonemic rats and examined whether MK-801 could prevent the increase in ammonia level in these organelles.

**Figure 2:**
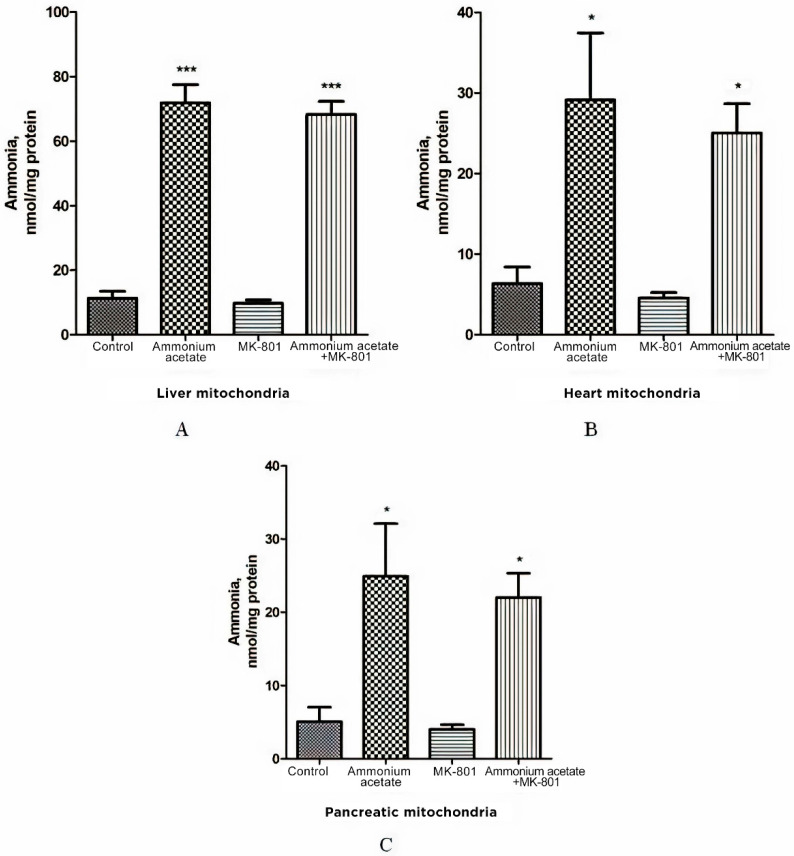
Effect of injection of ammonium acetate and/or MK-801 on ammonia concentration in mitochondria isolated from rat liver (**A**), heart (**B**), and pancreas (**C**). Immediately after decapitation, the tissues were removed, and mitochondria were isolated and assayed for ammonia levels, as indicated in Materials and Methods. Other experimental conditions were the same as in Figure 1 legend. The results are presented as mean ± SEM. Values significantly different from the control group are designated with one (*) and three (***) asterisks: * *p* < 0.05, *** *p* < 0.001 (Student’s *t*-test).

As shown in Figure 2A, the ammonia level in liver mitochondria of animals receiving a sublethal dose of ammonium acetate was six times higher than in control rats (71.9 ± 5.6 nmol/mg protein, and 11.3 ± 2.18 nmol/mg protein, respectively, *p* < 0.001), while the concentration of ammonia in the mitochondria of the heart and the pancreas increased approximately 4.5 fold and reached 29.13 ± 8.3 nmol/mg protein and 24.9 ± 7.2 nmol/mg protein, respectively, (*p* < 0.05, Figure 2B,C).

Despite the existing downward trend, the use of the injection that contained only MK-801 did not cause a significant decrease in ammonia concentration in all types of mitochondria compared to the control (Figure 2). NMDAR blockade with MK-801 also failed to affect ammonia content in mitochondria of hyperammonemic animals, and its level remained as high as in the ammonia group (Figure 2).

The lack of the effect of MK-801 on the increase in ammonia levels in all types of mitochondria indicates that NMDAR activation does not play a key role in ammonia accumulation in these cell structures. This is in agreement with data showing that intramitochondrial steady-state ammonia concentration in hyperammonemia is dependent on many factors, especially the rate of transport from the blood and endogenous ammonia production by ammonia-forming reactions [66,67].

### 3.3. The Effect of MK-801 on Ammonium-Dependent Disturbance of the Balance between Oxidant and Antioxidant Systems in Non-Neuronal Tissues

Given the positive correlation between levels of ammonia accumulated in brain mitochondria and oxidative stress [12], which is completely suppressed by MK-801 [22,51], we assessed whether ammonia accumulation in mitochondria isolated from NMDAR-equipped peripheral tissues, as well as in erythrocytes, would be associated with oxidative stress. We measured the activity of antioxidant enzymes SOD, catalase, GP, and GR in erythrocytes and mitochondria of the liver, heart, and pancreas of hyperammonemic animals. Additionally, we assessed whether MK-801 could affect defense-related enzymes under these conditions.

#### 3.3.1. The Effect of MK-801 on the Enzyme Activity of Superoxide Dismutase, Catalase, Glutathione Peroxidase, and Glutathione Reductase in Erythrocytes of Hyperammonemic Rats 

**Figure 3:**
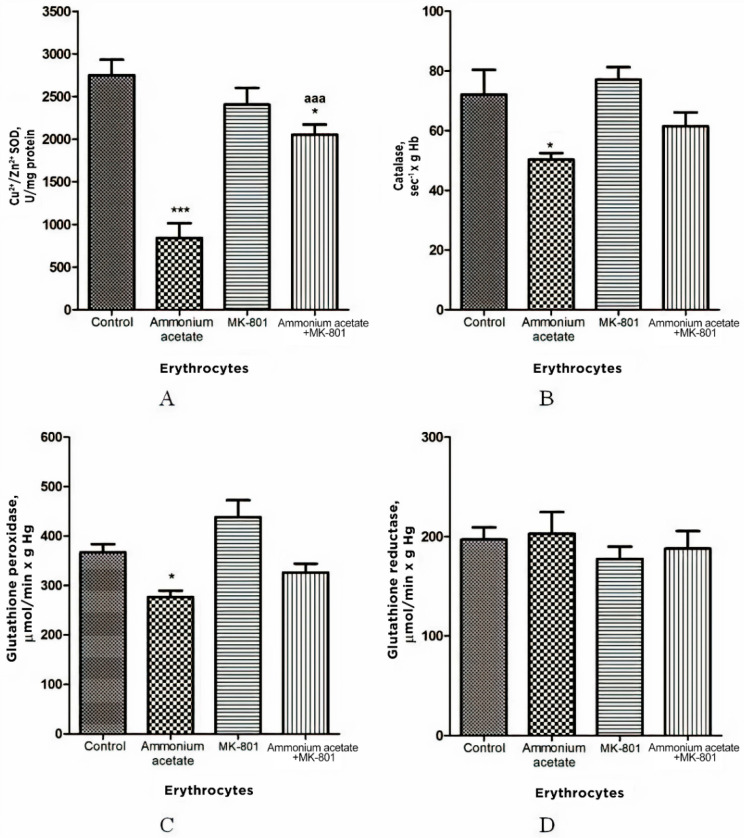
Activities of antioxidant enzymes superoxide dismutase (**A**), catalase (**B**), glutathione peroxidase (**C**), and glutathione reductase (**D**) in erythrocytes of animals in different experimental groups. Experimental design was the same as in Figure 1 legend. Erythrocyte enzyme activity was measured as indicated in Materials and Methods. The results are presented as mean ± SEM. Values significantly different from control are designated with one (*) and three (***) asterisks: * *p* < 0.05, *** *p* < 0.001 (Student’s *t*-test). ^a^ significant differences, compared with the ammonia group. ^aaa^ *p* < 0.001 (with the Bonferroni correction for multiple comparisons).

As shown in Figure 3, ammonium acetate injection leads to decreased activity of antioxidant enzymes in erythrocytes. The enzyme activity of SOD, catalase, and GP was decreased by 69% (*p* < 0.001), 30% (*p* < 0.05) and 25% (*p* < 0.05), respectively. GR activity remained unaltered in ammonia intoxication.

The injection that contained only MK-801 had no effect on the activity of any enzymes measured. In rats treated with MK-801 and ammonium acetate combined, only SOD activity was increased by 144% (*p* < 0.001), compared with the ammonium group, while the enzyme activity of GR, catalase, and GP did not significantly change (Figure 3).

From these results, it can be deduced that MK-801 partially prevented an ammonia-induced decrease in antioxidant enzyme activity in erythrocytes, and therefore, although prooxidant action of ammonia in erythrocytes is not exclusively dependent on NMDAR-mediated effects, activation of this receptor is an essential step in initiating oxidative stress in the erythrocytes of hyperammonemic animals.

#### 3.3.2. The Effect of MK-801 on the Enzyme Activity of Superoxide Dismutase, Catalase, Glutathione Peroxidase, and Glutathione Reductase in Liver Mitochondria of Hyperammonemic Rats

**Figure 4:**
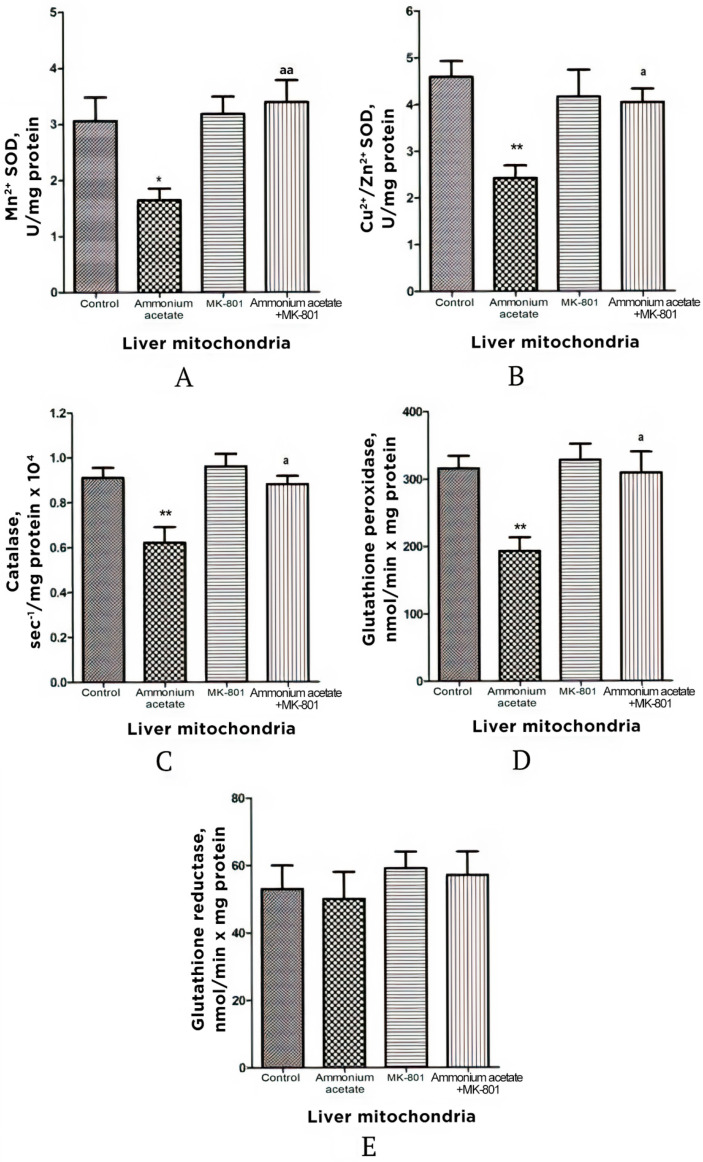
Activities of antioxidant enzymes superoxide dismutase (Mn^2+^ (**A**) and Cu^2+^, Zn^2+^ (**B**) isoforms), catalase (**C**), glutathione peroxidase (**D**), and glutathione reductase (**E**) in liver mitochondria of animals in different experimental groups. Experimental design was the same as in Figure 1 legend. The enzyme activity was measured as indicated in Materials and Methods. All data are shown as mean ± SEM. Values significantly different from the control group are designated with one (*) and two (**) asterisks: * *p* < 0.05, ** *p* < 0.01 (Student’s *t*-test). ^a^ significant differences when compared with ammonia group. ^a^ *p* < 0.05, ^aa^ *p* < 0.01 (with the Bonferroni correction for multiple comparisons).

In comparison with control, the enzyme activity of SOD (Mn^2+^ and Cu^2+^, Zn^2+^ isoforms), catalase, and GP in liver mitochondria of hyperammonemic rats was significantly lower, by 46–47% (both isoforms *p* < 0.05–0.01), 32% (*p* < 0.01), and 39% (*p* < 0.01), respectively, while GR activity remained unchanged (Figure 4).

The injection that contained only MK-801 had no effect on the activity of any enzymes measured, and values for the enzyme activity were close to the control limits (Figure 4) However, this injection completely prevented an ammonia-induced decrease in SOD, catalase, and GP activities, supporting the idea that, as in the mitochondria of the brain [22,51], the prooxidant effects of ammonia in liver mitochondria are mediated by NMDAR activation (Figure 4).

#### 3.3.3. Effects of Acute Ammonia Intoxication on Superoxide Radical and Hydrogen Peroxide Production by Liver Mitochondria

Oxidative stress in cells is caused by an imbalance between reactive oxygen species (ROS) formation and neutralization by antioxidants [68]. Since the reduced activity of antioxidant enzymes found in the liver mitochondria of hyperammonemic animals may trigger oxidative stress development, we evaluated whether acute ammonia intoxication increases the formation of other oxidative stress components—superoxide radical (O_2_^•−^) and hydrogen peroxide (H_2_O_2_)—in liver mitochondria and how MK-801 affects this process.

**Figure 5:**
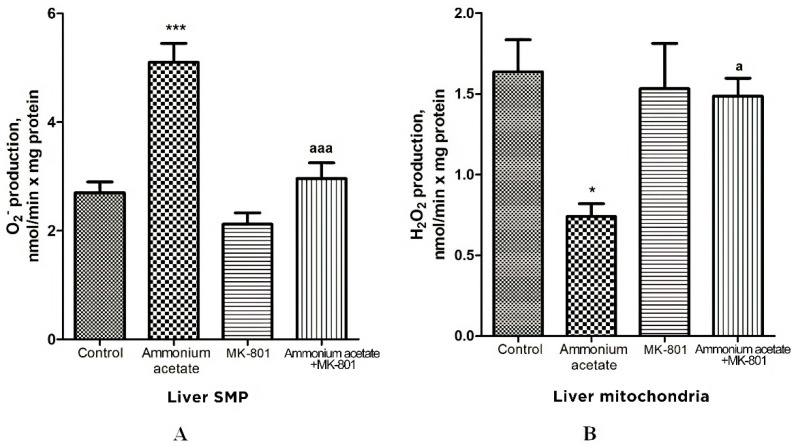
Effects of injection of ammonium acetate and/or MK-801 on O_2_^•−^ (**A**) and H_2_O_2_ (**B**) production by liver SMP and mitochondria. Experimental design was the same as in Figure 1 legend. The rates of O_2_^• −^ and H_2_O_2_ formation were determined as indicated in Materials and Methods. All data are shown as mean ± SEM. Values significantly different from the control group are designated with one (*) and three (***) asterisks: * *p* < 0.05, *** *p* < 0.001 (Student’s *t*-test). ^a^ significant differences, compared with ammonia group. ^a^ *p* < 0.05, ^aaa^ *p* < 0.001 (with the Bonferroni correction for multiple comparisons).

The rate of O_2_^•−^ generation by control liver SMP was 2.7 ± 0.2 nmol/min per mg protein (Figure 5) and was increased by 88% (*p* < 0.001) in SMP from rats injected with ammonium acetate. MK-801 did not affect O_2_^•−^ formation rate per se but completely prevented an ammonia-induced increase in O_2_^•−^ production (Figure 5), indicating that it was mediated by NMDA receptor activation.

In contrast to O_2_^•−^ production, the rate of H_2_O_2_ generation by liver mitochondria of hyperammonemic rats was inhibited by 55% (Figure 5B, *p* < 0.05), compared with control. This inhibition is supposedly dependent on the presence of a number of specific factors that inhibit H_2_O_2_ formation by mitochondria in rat models of hyperammonemia [69,70]. A single injection of MK-801 had no effect on H_2_O_2_ formation, whereas an ammonia-induced decrease in H_2_O_2_ production in liver mitochondria was completely prevented by NMDAR blockade with MK-801. Taken as a whole, these results confirm the accepted view that due to heteromeric assembly NMDARs mediate a wide range of signaling processes [71]. Additionally, they suggest that the factors regulating the rate of H_2_O_2_ and O_2_^•−^ formation by mitochondria are under NMDAR functional diversity [71].

#### 3.3.4. The Effect of MK-801 on Antioxidant Enzyme Activities and Superoxide Radical and Hydrogen Peroxide Production in Pancreas Mitochondria of Hyperammonemic Rats

**Figure 6:**
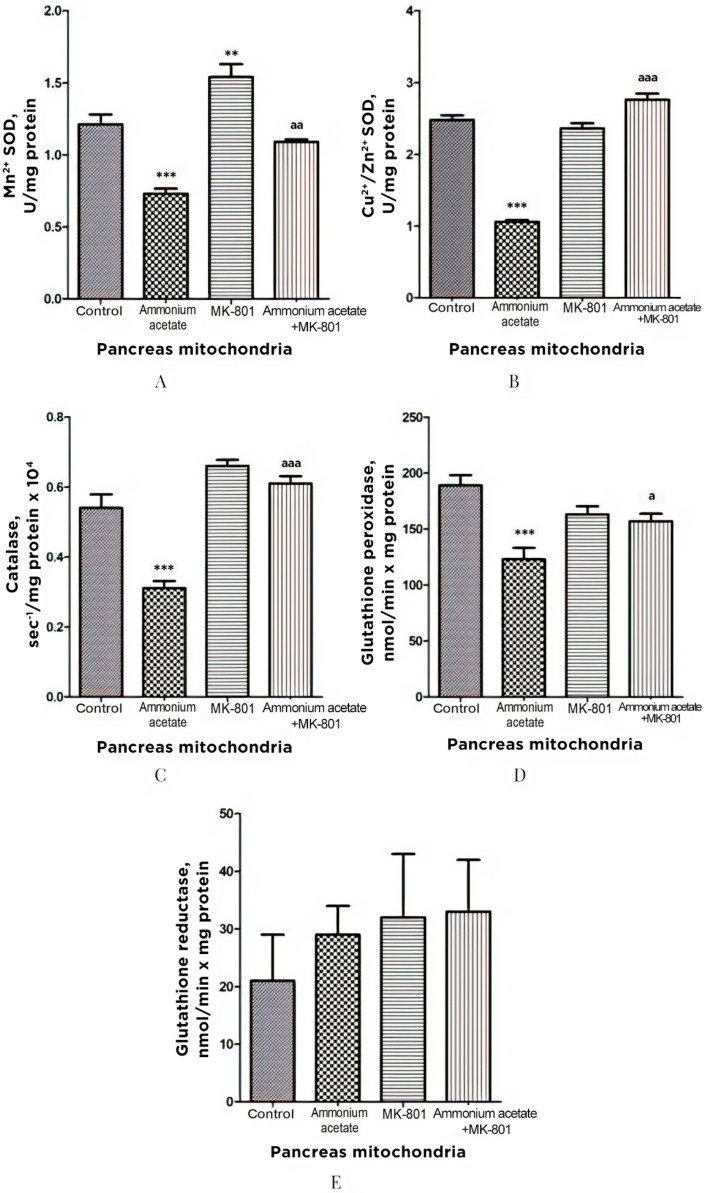
Effects of injection of ammonia and/or MK-801 on the activity of antioxidant enzymes superoxide dismutase (Mn^2+^ (**A**) and Cu^2+^, Zn^2+^ (**B**) isoforms), catalase (**C**), glutathione peroxidase (**D**), and glutathione reductase (**E**) in pancreatic mitochondria. Experimental design was the same as in Figure 1 legend. The enzyme activity was determined as indicated in Materials and Methods. The results are represented as mean ± SEM. Values significantly different from the control group are designated with two (**) and three (***) asterisks: ** *p* < 0.01, *** *p* < 0.001 (Student’s *t*-test). ^a^ significant differences, compared with ammonia group. ^a^ *p* < 0.05, ^aa^ *p* < 0.01, ^aaa^ *p* < 0.001 (with the Bonferroni correction for multiple comparisons).

The effect of ammonia on antioxidant and prooxidant status was similar in pancreas and liver mitochondria. Pancreatic Mn^2+^-SOD, Cu^2+^,Zn^2+^-SOD, catalase, and GP activities were reduced by 40, 57, 42.5, and 35%, respectively, after ammonium acetate injection (Figure 6). MK-801 alone had either no or minimal effect on the activity of enzymes measured but significantly increased Mn^2+^-SOD activity, compared with control (27%, *p* < 0.01).

Blocking NMDA receptors with MK-801 completely prevented the development of the ammonia-induced decrease in the activity of these enzymes. GR activity remained unaltered even after injection of ammonium acetate, MK-801, or ammonium acetate plus MK-801 (Figure 6). These results indicate that the ammonia-induced reduction in the enzyme activity of both SOD isoforms, catalase, and GP, in pancreas mitochondria, as in liver mitochondria, is mediated by activation of NMDA receptors.

**Figure 7:**
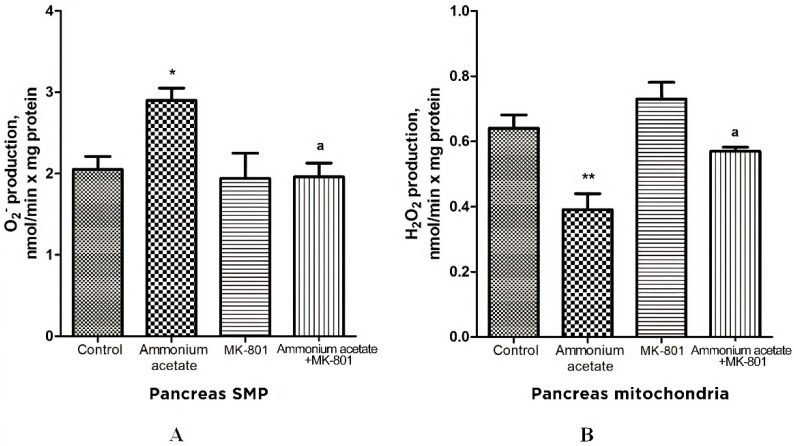
The effect of ammonium acetate and MK-801 injection on the rate of O_2_^•−^ (**A**) and H_2_O_2_ (**B**) formation in rat pancreas SMP and mitochondria. Experimental design was the same as in Figure 1 legend. The rates of O_2_^•−^ and H_2_O_2_ formation were determined as indicated in Materials and Methods. The results are represented as mean ± SEM. Values significantly different from the control group are designated with one (*) and two (**) asterisks: * *p* < 0.05, ** *p* < 0.01, (Student’s *t*-test). ^a^ significant differences, compared with ammonia group. ^a^ *p* < 0.05 (with the Bonferroni correction for multiple comparisons).

As shown in Figure 7A, O_2_^•−^ production was increased by 41% in pancreas SMPs of rats injected with ammonium acetate, compared with control. MK-801 injected alone did not affect that process but, as in liver SMP, completely prevented the ammonia-induced increase in O_2_^•−^ formation, indicating that it was mediated by activation of NMDA receptors.

As shown in Figure 7B, ammonia injection leads to a significant decrease (approximately by 40%, compared with control) in H_2_O_2_ formation in pancreas mitochondria. MK-801 alone induced a small, insignificant increase in the rate of H_2_O_2_ formation, compared with control.

The ammonia-induced decrease in H_2_O_2_ formation was completely prevented by the previous injection of MK-801. Thus, changes in the rate of H_2_O_2_ formation by mitochondria of the pancreas resemble those that occurred in the liver and were mediated by activation of NMDA receptors.

#### 3.3.5. The Effect of MK-801 on Activities of Antioxidant Enzymes, Production of Superoxide Radical and Hydrogen Peroxide in Heart Mitochondria of Hyperammonemic Rats 

**Figure 8:**
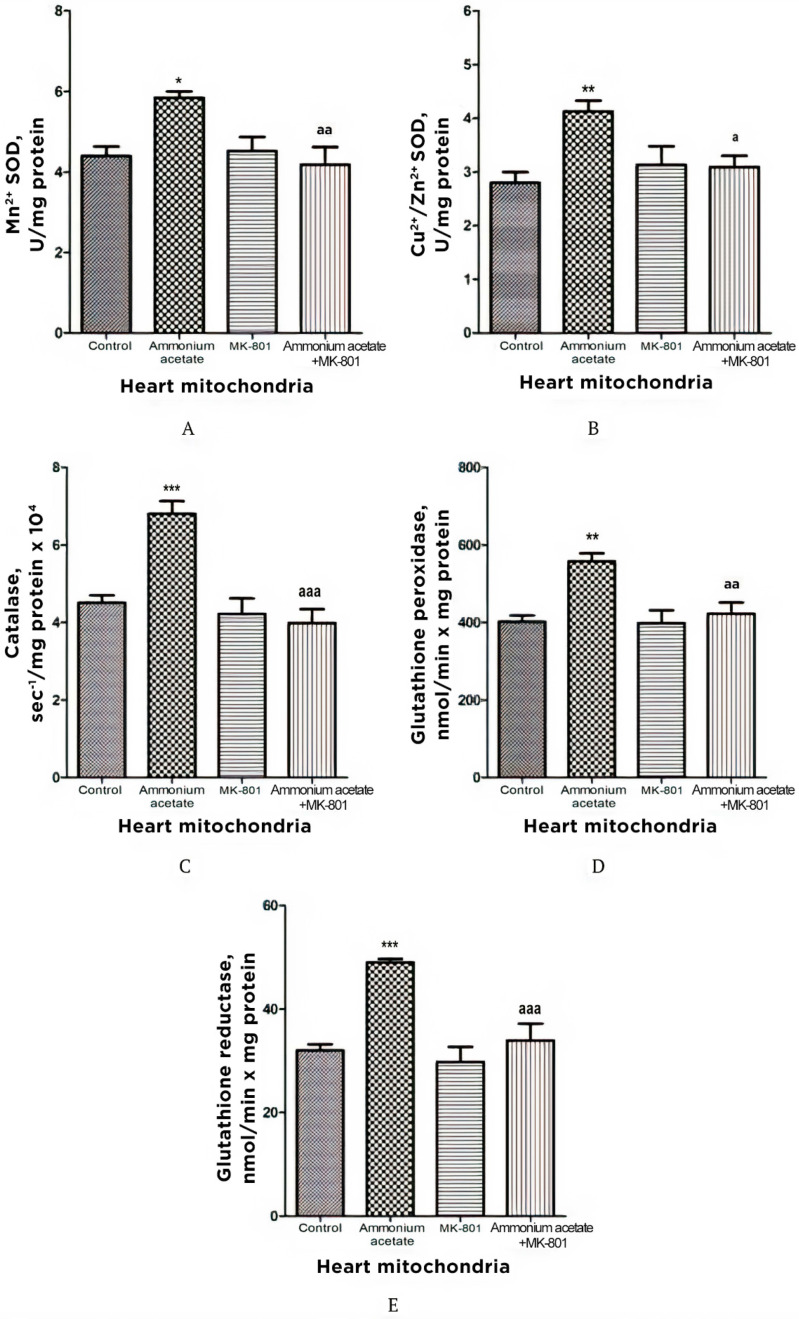
Effects of injection of ammonia and/or MK-801 on the activity of antioxidant enzymes superoxide dismutase (Mn^2+^ (**A**) and Cu^2+^, Zn^2+^ (**B**) isoforms), catalase (**C**), glutathione peroxidase (**D**), and glutathione reductase (**E**) in heart mitochondria. Experimental design was the same as in Figure 1 legend. Enzyme activities were determined as indicated in Materials and Methods. The results are represented as mean ± SEM. Values significantly different from the control group are indicated by one (*), two (**) and three (***) asterisks: * *p* < 0.05, ** *p* < 0.01, *** *p* < 0.001 (Student’s *t*-test). ^a^ significant differences, compared with the ammonia group. ^a^ *p* < 0.05, ^aa^ *p* < 0.01, ^aaa^ *p* < 0.001 (with the Bonferroni correction for multiple comparisons).

The effect of ammonium on the same enzymes in the heart mitochondria was opposite to the effect observed in erythrocytes, as well as in the liver, and pancreas mitochondria. Mn^2+^-SOD, Cu^2+^,Zn^2+^-SOD, catalase, GP, and GR activities in heart mitochondria of hyperammonemic rats increased by 33, 47.5, 51, 38.5, and 53%, respectively, compared with control (Figure 8). NMDAR block with MK-801 completely prevented the ammonia-induced increase in the activity of these enzymes.

**Figure 9:**
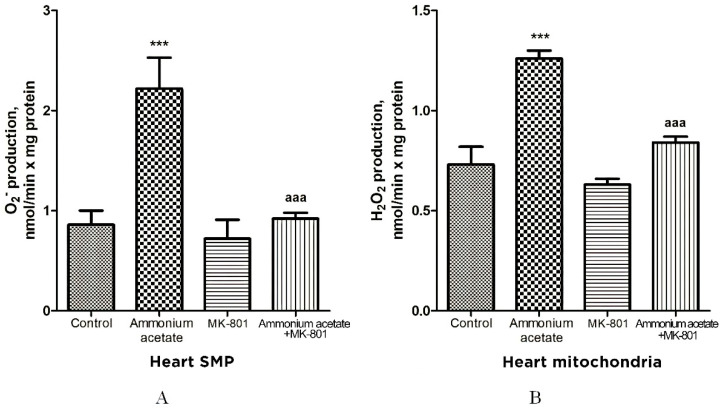
The effect of ammonium acetate and MK-801 injection on the rate of O_2_^•−^ (**A**) and H_2_O_2_ (**B**) formation in rat heart SMPs and mitochondria. Experimental design was the same as in Figure 1 legend. The rates of O2^•−^ and H_2_O_2_ formation were determined as indicated in Materials and Methods. The results are represented as mean ± SEM. Values significantly different from the control group are designated with three (***) asterisks: *** *p* < 0.001 (Student’s *t*-test). ^a^ significant differences, compared with ammonia group. ^aaa^ *p* < 0.001 (with the Bonferroni correction for multiple comparisons).

The rate of O_2_^•−^ generation by heart SMPs from rats injected with ammonium acetate was increased by 158% (*p* < 0.001), compared with control. MK-801 did not affect the rate of O_2_^•−^ formation per se but completely prevented an ammonia-induced increase in O_2_^•−^ production (Figure 9A), indicating that it was mediated by NMDA receptor activation.

Similarly (although the effect was less pronounced), ammonium acetate affected the rate of H_2_O_2_ formation (Figure 9B). The rate of H_2_O_2_ formation in the heart mitochondria of hyperammonemic animals increased by 72% in comparison with the control.

Acute injection of MK-801 did not change this parameter, while combined administration of MK-801 and ammonium acetate reduced the rate of H_2_O_2_ formation to the control level.

Our results confirm and expand on the basic concept of increased ROS production as a compensatory mechanism (albeit bordering on pathology) aimed at cardioprotection [72,73,74,75,76] and of ammonium-dependent enhanced generation of O_2_^•−^ and H_2_O_2_ as an apparent adaptive response of cardiac tissue, regulated by NMDA receptor-mediated signaling, which can also be either protective or destructive [77].

## 4. Discussion

Liver disease, as well as hepatic encephalopathy (HE) as its major complication, remains one of the leading causes of death in the world [78]. Although extensive research has been carried out on explaining this complicated disease, no single study exists that adequately describes the pathogenesis of HE.

According to a prevailing hypothesis, hyperammonemia is an important causative factor in hepatic encephalopathy, due to impaired liver function or after portocaval shunt, allowing blood ammonia to enter the brain unhindered, where it disrupts the normal brain function [3,4,5].

Multiple mechanisms that potentially cause neuronal cell death in hyperammonemia have been identified [6]. It has been convincingly demonstrated that glutamate, as the main neurotransmitter, plays a key role in triggering a cascade of pathological reactions in hyperammonemic encephalopathy, primarily due to hyperactivation of NMDAR [18,19,20,21,22,23], ultimately leading to brain pathology [7,67,79,80].

However, the recent discovery of the distribution of functional NMDARs outside the CNS in various peripheral tissues [24,25,26,27] supports the hypothesis that HE is a multisystem disease, and multiorgan failure is a syndrome that represents a pathophysiologic pathway leading to organ dysfunction [28,81] and, therefore, is important to be reviewed.

Many studies demonstrated that oxidative stress is one of the leading links in the pathological cascade triggered by overactive NMDAR signal transduction pathways in the brain of hyperammonemic animals [15,22,45,46] and HE patients [47,48,82], but the exact relationship between NMDA receptors present in peripheral non-neuronal tissues and oxidative stress involved in the pathogenesis of multiorgan pathology in HE [28,49] has not yet been completely understood.

It is also worthy of note that there is insufficient information so far to draw conclusions as to whether or not non-neuronal organs are sensitive targets of ammonia toxicity in experimental animals and even in HE patients. Given this, our research efforts narrowed the focus to explore the distribution of ammonia in erythrocytes and mitochondria of peripheral tissues containing NMDARs including pancreas, heart, and liver of hyperammonemic rats and whether the ammonia-induced oxidative stress is mediated by these receptors.

We revealed that the ammonia level in erythrocytes was considerably increased in rats injected with ammonium acetate (14 fold, *p* < 0.001, Figure 1). When MK-801 was injected together with ammonium acetate, the ammonia concentration in erythrocytes remained higher than that in control but decreased significantly by 19.8% (*p <* 0.05), compared with those in animals of the ammonia group. These results show that MK-801 slightly prevents the increase in ammonia concentration of blood plasma and erythrocytes in hyperammonemic rats and, therefore, cannot significantly reduce the ammonia load in other cells and their intracellular compartments.

The ammonia gradient detected between plasma and erythrocytes (ammonia concentration is higher in erythrocytes than in plasma) in control animals (Figure 1) corresponds to the normal distribution of ammonia in blood [65]. The persistence of this gradient with a significant accumulation of ammonia in erythrocytes in hyperammonemic animals indicates that erythrocytes can serve as a temporary depot for ammonia. However, rats’ mature erythrocytes lack glutamine synthetase and glutamate dehydrogenase, which could reduce the toxic load in these cells by converting ammonia into glutamine and glutamate, respectively. This makes these cells very susceptible to the toxic effects of ammonia, which can manifest through more severe ammonia-related complications, including oxidative stress and those affecting blood oxygen transport and hemoglobin function [83,84,85].

Our finding demonstrated three antioxidant enzymes—SOD, catalase, and GP—that decreased in rat erythrocytes after ammonium acetate injection (69% (*p* < 0.001), 30% (*p* < 0.05) and 25% (*p* < 0.05), respectively) without change in GR. MK-801 did not prevent significantly ammonia-induced changes in GR, catalase, and GP activities, and only SOD activity was increased by 144% (*p* < 0.001), compared with the ammonium group (Figure 3).

These results show that, although prooxidant action of ammonia in erythrocytes is not due solely to NMDAR-mediated effects, activation of this receptor is important for initiating oxidative stress in the erythrocytes of hyperammonemic animals.

Erythrocytes are the only cells that transport oxygen and maintain aerobic utilization of glucose in tissue. It is recognized that the role of these cells in tissue oxygen delivery depends on their intracellular metabolism, primarily via energy metabolism and antioxidant status [86,87,88]. These processes result in the formation of allosteric effectors tht modulate hemoglobin oxygen affinity [89], which determines the capacity of Hb to bind oxygen in the lungs as much as possible and release the necessary amount of it to the tissues [86].

However, it is currently still not known whether the toxic effects of ammonia accumulated in erythrocytes induce changes seen to glycolysis, the oxygen affinity of hemoglobin [90], as well as the accelerated autoxidation of Hb resulting in the formation of methemoglobin that is incapable of carrying oxygen to tissues [82], which ultimately leads to erythrocyte dysfunction [35,91] and their premature senescence [92]. For this reason, the need to clarify this point becomes a matter of urgency.

MK-801 did not prevent an increase in the level of ammonia in mitochondria isolated from the liver, pancreas, and heart of hyperammonemic animals, and its level remained as high as in the ammonia group (Figure 2). This indicates that activation of NMDARs in these tissues, or more specifically, activation of NMDAR signal transduction pathways has no impact on ammonia accumulation in mitochondria. This is in agreement with the data showing that in hyperammonemia, the steady-state intramitochondrial ammonia concentration depends on many factors [43,93], especially on the rate of its transport from the blood to mitochondria; this relies particularly on the availability of mitochondrial aquaporin-8, a membrane channel, permeable to ammonia [94,95], on ammonia-forming endogenous reactions in mitochondria [66,67] and glutamine synthetase activity [96].

Given the positive correlation between levels of ammonia accumulated in brain mitochondria and oxidative stress [12], which is completely suppressed by MK-801 [22,51], we assessed whether ammonia accumulation in mitochondria isolated from NMDAR-equipped peripheral tissues is associated with NMDAR-mediated oxidative stress.

As shown in Figure 5A and Figure 7A, O_2_^•−^ production rate in SMPs isolated from the liver and pancreas of ammonium acetate-treated animals was significantly higher than in the control group, and MK-801 completely prevented an ammonia-induced increase in O_2_^•−^ production in SMPs of these tissues (Figure 5A, Figure 7A and Figure 9A), indicating that this process was mediated by NMDA receptor activation.

In contrast to O_2_^•−^ production, the rate of H_2_O_2_ generation by liver and pancreas mitochondria of hyperammonemic rats was inhibited by 55% (Figure 5B, *p* < 0.05) and 40% (Figure 7B, *p* < 0.01), respectively, compared with control. MK-801 treatment did not affect H_2_O_2_ formation in liver and pancreas mitochondria, whereas an ammonia-induced decrease in H_2_O_2_ production in these tissues was completely prevented by NMDAR blockade with MK-801.

Considering the fact that effects of MK-801 on the rate of H_2_O_2_ formation in liver and pancreas mitochondria were similar to those implicated in the brain mitochondria of hyperammonemic animals [51], we can assume that, in addition to the numerous known factors that inhibit the production of H_2_O_2_ in mitochondria [97], the ammonia-related reduction in H_2_O_2_ production in liver and pancreas mitochondria could be due to a decrease in NADH availability [69,70], as evident from a significant increase in the mitochondrial NAD/NADH ratio [10,56], as well as due to a significant decrease in hepatic (Figure 4) and pancreatic (Figure 6) activities of Mn^2+^-SOD localized in the mitochondrial matrix.

Taken altogether, in addition to a decrease in the activity of matrix SOD, the activity of its other isoform, Cu^2+^, Zn^2+^-SOD, localized in the intermembrane space, as well as activities of catalase and GP in mitochondria isolated from the liver (Figure 4) and pancreas of hyperammonemic rats were significantly reduced, compared with control, and MK-801 completely prevented an ammonia-induced decrease in the activities of all of these enzymes. Therefore, it might be concluded that the pro-oxidant effects of ammonia in liver and pancreas mitochondria are mediated by NMDAR activation.

Ammonia had the opposite effect on these indicators in the heart. O_2_^•−^ and H_2_O_2_ formation by heart mitochondria after ammonium acetate injection was almost double that of control (Figure 9A,B). Acute injection of MK-801 did not change this parameter, while combined administration of MK-801 and ammonium acetate reduced the rate of O_2_^•−^ and H_2_O_2_ formation to the control level (Figure 9B), indicating that generation of these highly reactive compounds in heart mitochondria was mediated by NMDA receptor activation.

With a parallel increase in the rate of O_2_^•−^ (Figure 9A) and H_2_O_2_ production (Figure 9B), a significant increase in the activities of Mn^2+^-SOD, Cu^2+^,Zn^2+^-SOD, catalase, GP, and GR was observed in the heart mitochondria of hyperammonemic rats. Blocking NMDAR with MK-801 completely prevented an ammonia-induced increase in the activity of these enzymes.

The differential response of the heart to oxidative stress and upregulation of key antioxidant enzymes are consistent with those observed under oxidative stress conditions in other studies [98]. The heart is the most adaptive organ, and increased activities of antioxidant enzymes in heart mitochondria could reflect a compensatory adaptive response to the prooxidant effect of ammonia [99,100].

The multidirectional effects of NMDAR activation on the rate of O_2_^•−^ and H_2_O_2_ formation in the liver, pancreas, and heart mitochondria of hyperammonemic animals confirm the well-known point of view that the heteromeric nature of NMDARs allows a rich diversity in receptor signaling properties [71] and that effects of NMDAR activation can have a dual nature, showing both toxic and survival-promoting effects [71].

In general, these findings for the first time demonstrate a precise relationship between ammonia-inducing oxidative stress in mitochondria of peripheral non-neuronal tissues and hyperactivation of NMDA receptors present in these tissues, including the liver, heart, and pancreas, as well as in erythrocytes.

It is known that overproduction of ROS in mitochondria can disrupt both the function of mitochondria and other cellular components and usually precedes the development of most human diseases [101]. Thus, it is clear that identifying the causes that lead to a violation of oxidative phosphorylation, inducing an increase in ROS production in mitochondria of non-neuronal tissue in hyperammonemic conditions, will help us understand the mechanisms underlying multiple organ failure, and expand our view of this disease.

It is also clear that dysfunction of erythrocytes during ammonia-induced oxidative stress is associated with the development of multiple organ pathology in HE. Surely, tissue oxygenation depends on erythrocytes that carry oxygen to cells but also on a wealth of complex regulatory mechanisms, including a precise functional relationship of three main systems implicated in oxygen transport, such as the cardiovascular, respiratory systems, and blood [102,103]. However, despite the importance of the main systems for delivering oxygen to tissues—the lungs, heart, blood vessels carrying blood to various organs—the final step in a normal oxygen supply to the tissues, where gas exchange occurs, is a function of erythrocytes.

We believe that careful examination and reversal of ammonia-related metabolic/energetic changes in erythrocytes modulating hemoglobin oxygen affinity will undoubtedly stimulate new directions in research and help identify additional risk factors for poor prognosis related to tissue hypoperfusion and multiple organ hypoxia [104,105,106,107] in patients with liver failure and, especially, in elderly patients with age-related erythrocyte metabolic disorders [108].

Altogether, the results obtained show that HE is multifactorial in its etiology and that multiorgan oxidative stress might be an integral part of a disease process, and therefore, the toxic effects of ammonia in HE, enhanced by NMDAR, may be more generalized and harmful than it was previously believed.

## Data Availability

Data are contained within this article.
